# A method to predict patient‐specific table coordinates for quality assurance in external beam radiation therapy

**DOI:** 10.1002/acm2.12428

**Published:** 2018-08-07

**Authors:** Daniel L. Saenz, Nestor Rodrigo Astorga, Neil Kirby, Mohamad Fakhreddine, Karl Rasmussen, Sotirios Stathakis, Niko Papanikolaou

**Affiliations:** ^1^ University of Texas Health San Antonio San Antonio TX USA

**Keywords:** external beam radiotherapy, patient safety, quality assurance

## Abstract

**Purpose:**

While external beam radiotherapy treatment planning determines nearly every mechanical and dosimetric parameter of the linear accelerator (LINAC), the table coordinates in all three dimensions are generally unknown until initial patient setup at the LINAC. Knowing these parameters in advance could help verify the direction of patient shifts and prevent wrong‐site errors. This study aims to determine the feasibility and accuracy of table coordinate prediction for indexed immobilization devices.

**Methods:**

A total of 303 table coordinates were predicted for patients on Varian and Elekta linear accelerators with immobilization devices including Orfit mask with baseplate, wingboard, breastboard and BodyFix. Predictions were made for all three spatial dimensions except for Body Fix setups due to the lack of a radiographically apparent indexing‐related landmark. Coordinates were predicted by measuring baseline table coordinates in all dimensions at specified landmark positions.

**Results:**

Predictions were accurate within 2 cm for 86% of coordinates (71% within 1 cm). Table coordinates were predicted most accurately for head and neck patients with a base plate and the most difficult prediction was in the lateral direction for breastboard patients.

**Conclusions:**

With proper indexing, table coordinates can be predicted with reasonable accuracy. The data suggest an action of level of 2 cm with certain exceptions for specific immobilization devices and directions.

## 1 INTRODUCTION

Patients undergoing radiation therapy are commonly localized by the use of reference marks made at the time of patient simulation (tattoos) which are subsequently identified in the treatment planning process. If another point is used for the treatment isocenter, shifts are calculated to be applied at the time of treatment. When first positioned at the linear accelerator (LINAC), the patient's reference marks are aligned to the LINAC lasers with a treatment table capable of undergoing translations in three dimensions and/or rotations in one or more dimensions. Any shifts are applied by mathematically adding and subtracting shift magnitudes to the observed table coordinates. The isocenter is checked by a second therapist before initializing verification imaging following institutional policy. Such imaging can verify field shapes relative to patient anatomy and/or use 3D anatomical detail to ensure accurate alignment with the planned isocenter. After approval of all imaging, the table coordinates are captured into the treatment field in the record‐and‐verify system for subsequent treatments. The treatment fields are then approved and locked with these table coordinates, and overrides are required in future fractions if the table coordinates ever deviate by more than a specified tolerance.^1^, ^2^, ^3^

The three‐dimensional position of the table is among many parameters set on the LINAC to ensure accurate patient setup and treatment. As described, however, the 3D table coordinates are not known until the patient is first positioned in the treatment room.^4^ Gantry angles, collimator angles, and jaw settings, etc are all meticulously planned and verified during pre‐treatment plan review, but typically only the table angle is set prior to treatment. Exact table coordinates are not necessarily required given the role of advanced image guidance as confirmation of accurate 3D anatomical localization. Prior knowledge of these table coordinates, however, may provide an early alert of an error if a mismatch is observed with observed table coordinates. Positioning errors could therefore be detected prior to exposure to imaging ionizing radiation.^5^, ^6^, ^7^ Mismatches could be resolved by a staff member before proceeding with imaging. Prior table coordinate knowledge also provides an independent isocenter verification not subject to errors in the image‐guidance process, prevents wrong direction patient shifts, and allows the medical physicist to review these coordinates during plan review as well.

Several studies have demonstrated the value of additional safety checks in patient positioning. Klein et al. discussed the possibility of wrong table shifts in 2005 in a study estimating the dosimetric impact of treatment errors and examined how a lateral table relative shift of +4.0 cm may be interpreted as the absolute coordinate itself.^8^ This study states that the issue could be remedied by “having the coordinates checked by the physicists or therapist staff pretreatment”. A 2009 report summarizing errors in the state of Pennsylvania showed that 32% of the reported events were from wrong location, wrong side, or wrong setup, and called for additional safety checks on location.^9^ Movements of the table from reference marks is an error‐prone step in the patient setup workflow, making up 10.8% of reportable radiation incidents analyzed in PHE Report No. 4.^10^ Common error pathways from Radiation Oncology Incident Learning System^®^ (RO‐ILS) showed that 74 of the 396 events were caused by either wrong shift instructions or wrong shift performed at treatment.^11^ Wrong site errors tend to be detected by port films, but this only applies on fractions where port films are taken. Studies have also examined the constriction of values for table coordinates for prevention of errors, but these data rely on baseline values only known after the first treatment fraction.^12^ The prediction of table coordinates may be a practical, valuable additional safety check that can be used without exposing the patient to additional ionization radiation. Moreover, this prediction completes the set of machine parameters set in the treatment fields prior to patient treatment, serving as an engineering control preventing treatment without higher approval, just as other machine geometrical parameters are treated (gantry angle, collimator angle, etc).

## 2 METHODS

Linear accelerators included in this study include the Elekta Versa HD, Varian 23EX, and Novalis Tx. Virtual simulation and treatment planning is performed in Philips Pinnacle^3^ version 9.16 and the record‐and‐verify system in use is MOSAIQ^®^. Table coordinates consist of parameters in the three spatial directions, referred to in this study as lateral (x), vertical (y), and longitudinal (z). The overall methodology is to identify baseline table coordinates (*T*_*x,0,*_ *T*_*y,0,*_ *T*_*z,0*_) corresponding to a coordinate (x_*0,*_ *y*_*0,*_ *z*_*0*_) in the tomographic slice of a landmark in an immobilization device and adjust them by planned patient isocenter patient shifts from this landmark point (*x*′, *y*′, *z*′). The longitudinal direction is the most difficult to predict, as it requires consistent indexing of immobilization devices.^13^ If this condition is met, the table longitudinal coordinate when a landmark slice in each immobilization device is set to isocenter is required. To predict the longitudinal table coordinate at the patient's isocenter, only the difference in slice position between this landmark and patient isocenter in the CT scan is needed (*z*′). This approach must be specific for each immobilization device and for each linear accelerator with a unique coordinate system.

The vertical dimension is more simply predicted by measuring the vertical distance between the planned isocenter and the treatment table in the CT‐simulation scan (*y*′). This process is facilitated by the inclusion of the treatment table in the treatment planning system. This has the additional advantage of verifying the correct vertical placement of the treatment table in the treatment planning process. It is required that the vertical table calibration is such that the table is at its nominal position, *T*_*y,0*_, when vertically set to isocenter.

Finally, the accuracy of the lateral coordinate depends on how laterally centered reference points are made at CT‐simulation and subsequently centered at treatment. If the patient is laterally centered, then an assumption can be made that the table is set at its nominal lateral position, *T*_*x,0*_, when the patient is aligned to his or her reference marks, and the final lateral coordinate is predicted by the lateral displacement of the isocenter coordinate from the reference coordinate (*x*′). The lateral table coordinate prediction becomes most difficult for patients reference marks are not placed at midline. Fortunately, most patients at our institution have reference marks placed in this manner, even those with a clear sidedness (e.g., right or left breast). However, this lateral coordinate prediction system is not applicable at sites such as extremities, where the patient is centered on the table, but both the reference and isocenter coordinates are laterally off‐center in an extremity.

**Figure 1:**
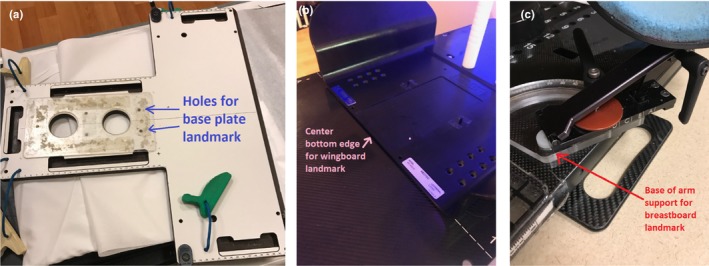
(a) Base plate for masked brain, head and neck patients. The inferior pair of holes is used as a longitudinal landmark for couch coordinate prediction. (b) Bottom edge of wingboard used as its landmark. (c) Base of arm support structure on breastboard is used as the landmark for couch coordinate prediction for patients immobilized with this device.

The prediction of the table coordinates in three dimensions is dictated by the immobilization device. Primarily, four devices are used at our institution: thermoplastic masks on a base plate for brain or head and neck treatments, a wingboard for chest and upper abdomen treatments, a breastboard for breast treatments, and finally a BodyFIX^®^ for most other treatments. All except BodyFIX^®^ have readily apparent landmarks radiographically apparent in the CT‐scans. The head and neck base plate (Orfit Industries, Belgium) has three pairs of holes, the lowest of which was identified as the landmark (Fig. 1). The bottom edge of the wingboard (Civco Radiotherapy, Orange City, IA, USA) was used as a landmark as was the bottom edge of the arm support structure for the breastboard (Qfix, Avondale, PA, USA) (Fig. 1). For the Bodyfix (Elekta, Stockholm, Sweden), there is no landmark to assist in the longitudinal prediction, so this parameter is not a candidate for patient‐specific prediction. It is possible to perform population‐based predictions for certain sites such as pelvis, but this is beyond the scope of this study.

**Figure 2:**
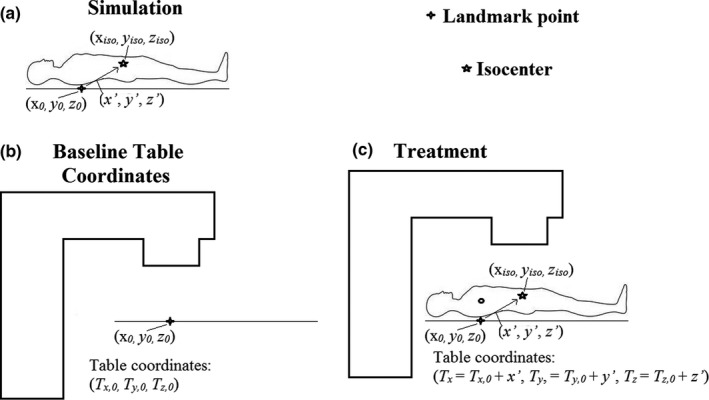
(a) Patient coordinates are shown according to the legend in the upper right. A landmark point is identified in the same slice as the landmark radiographically apparent in an immobilization device (not shown). The patient isocenter and translation vector between the points is also shown. (b) The same landmark point is setup to isocenter at the LINAC for the determination of baseline table coordinates. (c) For a specific patient, the table coordinates are determined by adding the translation vector to the baseline table coordinates.

The only remaining information needed is baseline table coordinate values at the landmark positions (the table laterally centered coordinate for lateral couch prediction, *T*_*x,0*_, the table vertical nominal value when raised to isocenter, *T*_*y,0*_, and the longitudinal positions of the table at each of the immobilization devices specified landmarks, *T*_*z,0*_). These values are summarized in Table 1. The displacement from these landmark values as measured in the treatment planning system are all that is required to predict the table coordinates (*T*_*x*_ =* T*_*x,0*_ + *x’*,* T*_*y*_, =* T*_*y,0*_ + *y’*,* T*_*z*_ =* T*_*z,0*_ + *z’*). Figure 2 outlines the geometry of the prediction method. Note that when using the 6 degree‐of‐freedom table top (HexaPod^TM^), the Precise Treatment System^TM^ table is raised 7 cm to situate the patient, requiring a additional 7 cm offset.

**Table 1:** Landmark table coordinate baselines for coordinate prediction. This table represents what the table coordinates are when setting the table laterally centered, vertically at isocenter, and longitudinally aligning lasers with the specified landmarks

Immobilization Device	Varian 21EX table landmark baseline coordinates (cm)			Novalis Tx table landmark baseline coordinates (cm)			Elekta VersaHD table landmark baseline coordinates (cm)		
	Vertical, T_y,0_	Lateral T_x,0_	Longitudinal T_z,0_	Vertical, T_y,0_	Lateral T_x,0_	Longitudinal T_z,0_	Vertical, T_y,0_	Lateral T_x,0_	Longitudinal T_z,0_
Head and neck base plate	100	100	120.4	100	100	126.5	0 (−7 with HexaPOD^TM^ in use)	0	57
Wingboard	100	100	117	100	100	117	0	0	47.8
Breastboard	100	100	122.6	n/a (no breast radiation on this linac)			0	0	31.7
Bodyfix	100	100	n/a	100	100	n/a	0	0	n/a

### 2.A Clinical verification

Current on‐treatment patient data was used to verify the prediction model. While the vast majority of table coordinates can be predicted, some coordinates cannot be predicted. These include the longitudinal coordinates for BodyFIX^®^patients and coordinates for patients treated with electrons. Patients to be treated with electron therapy were excluded due to the direct clinical verification of the light field on the skin being the most important consideration in our clinic, which overshadows the role of table coordinates for quality assurance. As an indication of the general applicability of this system, the total number of table coordinates required on a typical day was estimated to be 180 (60 patients with three dimensions of couch coordinates) of which all but 28 could be predicted (84%). Of those, 22 were expected BodyFIX^®^treatments (primarily pelvis, lumbar spine, or extremities) for which the longitudinal position is not predictable. Finally two patients were expected electron treatments (a total of six parameters not predictable).

Of the 303 couch coordinates from 100 patients studied, 84 were from breastboard setups, 66 from wingboard setups, 111 from mask and base plate setups, and 42 from BodyFIX^®^setups. These data include patients receiving 2D, 3D‐conformal, step and shoot IMRT, and VMAT. Data collected included the site, the indexing/immobilization device, the field ID and field names, isocenter name, the measured vertical distance between the isocenter and the table model in the planning system, the lateral coordinate of the reference marks and final isocenter positions, the longitudinal slice position of the landmark location and the final isocenter, and the captured table coordinates. Ongoing table coordinates used for subsequent fractions were not recorded as they are beyond the scope of this study. For each immobilization device, predictions were made separately for the Varian vs. Elekta vault (with or without use of HexaPOD^TM^). The differences between predicted and actual table coordinates were then analyzed in a spreadsheet.

## 3 RESULTS

Table 2 summarizes the accuracy of the prediction method for the total of 304 coordinates predicted. The results are specified for each immobilization device and each direction. Overall, 86% of the predictions were correct within 2 cm and the mean error was <0.1 cm. The standard deviation of the prediction errors was 1.47 cm. By immobilization device, the best results were obtained for head & neck patients, for which 82.0% of the time, parameters could be predicted within 1 cm. By direction, the vertical and longitudinal predictions were most accurate (>90% correct within 2 cm, and 88.5% correct within 1 cm for longitudinal). The standard deviation of the vertical prediction errors was 0.94 cm, while it climbs to 1.74 cm for longitudinal and to 2.28 cm for lateral.

**Table 2:** Accuracy of prediction method summarized by percentage of predictions accurate within 1 and 2 cm. Results are stratified by immobilization device, and by table dimension

	Percent of coordinates accurate within 1 cm (2 cm)			
	All dimensions (303)	Vertical (108)	Lateral (108)	Longitudinal (87)
All devices (303)	71.4% (86.2%)	78.9% (95.4%)	50.0% (70.4%)	88.5% (94.3%)
H & N (111)	82.0% (93.7%)	94.6% (100.0%)	67.6% (94.6%)	83.8% (86.5%)
Wingboard (66)	66.7% (86.4%)	68.2% (90.9%)	31.8% (68.2%)	100.0% (100.0%)
Breastboard (84)	54.8% (73.8%)	57.1% (89.3%)	21.4% (32.1%)	85.7% (100.0%)
Bodyfix (42) (no longitudinal)	83.7% (90.7%)	90.5% (100.0%)	76.2% (81.0%)	n/a

**Figure 3:**
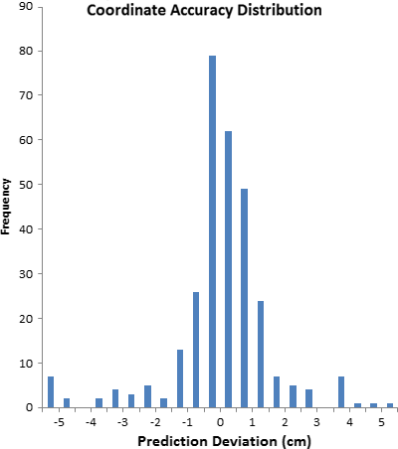
Distribution of the difference between predicted and actual couch coordinates. Data is across all directions and treatment units.

**Figure 4:**
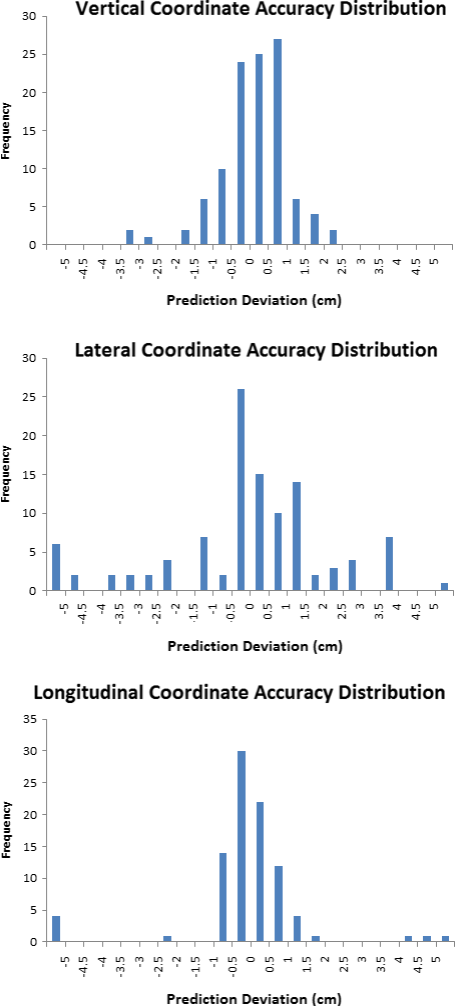
Distribution of the difference between predicted and actual couch coordinates, broken down in all three directions.

Figure 3 displays the distribution of prediction error across all patients, while Fig. 4 breaks down the distribution specific to dimension. The standard deviation in error was the largest in the lateral direction, and smallest in the vertical dimension. The maximum prediction error found in the study was 14.2 cm. However, this is an example of the outcome when a patient is indexed to a non‐standard location on the treatment table. Because this patient was setup using the head and neck base plate but was being treated to the mediastinum, the patient was setup to a different place on the table (for clearance reasons).

In this study, the use of six‐degree‐of‐freedom table tops was considered with two devices: the Elekta HexaPOD^TM^ and the BrainLab ExacTrac^®^ table top. The HexaPOD^TM^ from Elekta has a baseline lowered position when not in use and a raised position when in use. This load position required a consistent 7 cm offset to be introduced for all predictions. The BrainLab ExacTrac^®^ table top does not have a separate loading position, and no baseline adjustments were necessary. Table coordinates for patients setup with ExacTrac^®^ were predicted with no greater error than those with 3D corrections only (standard deviation in table prediction error of 0.36 cm vs. 0.39 cm). For HexaPOD^TM^ vs. ExacTrac^®^ specifically, 92.9% were predicted within 2 cm for HexaPOD^TM^ vs. 100% for ExacTrac^®^.

## 4 DISCUSSION

With an accuracy of 2 cm for the majority of cases, it could prove advantageous to add this prediction to pre‐treatment quality assurance procedures to verify isocenter prior to imaging. An action level of 2 cm could be implemented to require review by a physician or physicist when the final table coordinates differ by at least this amount. With 86% accuracy, this would not significantly increase the burden on the clinical workflow. In fact, immobilization device specific action levels could be instituted, as well as setting alternate action levels in each dimension.

Specific changes in workflow would vary amongst clinical practices, but can be understood through the following example. In current practice, the patient is aligned to reference marks, and any necessary shifts are applied by mathematically adding or subtracting shift magnitudes to the observed table coordinates. A second therapist verifies the shifts before proceeding to acquire port films. With table coordinate prediction, the role of the second therapist becomes to instead compare the observed table coordinates with those pre‐populated in the treatment field or plan document. At this point the verifying therapists will alert the physicist or physician as appropriate. The verification imaging and treatment would follow as normal, with the added efficiency that table coordinates no longer require capturing as in the current workflow. With coordinates known and pre‐populated in the record‐and‐verify system requiring overrides to bypass, the confirmation of table coordinates serves as more of an engineering control rather than a simple auto‐populated parameter which personnel can become immune to.

While image guidance such as kV planar and cone‐beam CT imaging can more precisely align an internal patient point to isocenter than table coordinates alone, this prediction model adds an important tool to improving patient safety in situations where image guidance fails. For instance, image guidance relies on the correct transfer of the isocenter DICOM coordinates from the treatment planning system eventually to the cone‐beam CT alignment software. Errors in this process can occur due to a number of causes including manual coordinate entry errors (incorrect sign, unit conversion mistakes, etc). Image‐guidance could then systematically align the patient incorrectly for every treatment fraction. The simultaneous comparison with table coordinates performed independently in the treatment planning system, prior to a coordinate entry error in the record‐and‐verify system, would be an important detection tool for wrong isocenter treatments. Table coordinate prediction could also help in other scenarios including concurrent treatments to two sites. A therapist may mistakenly believe a patient was planned with a single isocenter for setup efficiency when the patient was in fact planned with two isocenters. The pre‐population of table coordinates would result in distinct coordinates immediately apparent to a therapist prior to port filming or cone‐beam CT, reducing unnecessary imaging dose and reducing treatment times. In summary, this methodology in no way precludes or diminishes the necessity of cone‐beam CT image guidance, but serves as a supplementary safety verification.

Of those coordinates which were outside of 2 cm, the lateral dimension was the primary direction of error, mostly due to uncertainties on the lateral setup of a patient on the treatment couch for any site with any sidedness (e.g., breast). While patients most commonly have reference marks at midline, sites such as breast (which change patient pose by raising an arm) frequently require placing reference marks somewhere other than the lateral center of the table. This requires the table to be offset, leading to errors in the prediction. For sites such as head & neck, this lateral error was much smaller, suggesting an appropriately wider action for breast as opposed to sites with less lateral uncertainty such as head & neck. With the suggested action levels for a site like head & neck, physician or physicist intervention would have been required in no more than 14% of treatment validations. This would represent a small additional effort, and would additionally be expected to decrease re‐imaging with ionizing radiation in cases where the patient requires repositioning.

Another issue which arose during the analysis were instances when the landmark on the immobilization device was outside of the range of the CT scan. This is easily remedied, however, by finding baselines for multiple landmark positions on an immobilization device. A related problem and source of error in couch coordinate prediction is misidentification of landmark positions in the prediction process, which can lead to inaccurate predictions at the time of initial patient setup at the linear accelerator. Choosing landmarks on the immobilization devices at regular intervals corresponding to index locations on the table can help identify the source of these errors (e.g., if the deviation is exactly 10 cm, then it is likely due to misidentification of the landmark at the time of coordinate prediction).

In addition to being unable to predict longitudinal coordinates when devices without an apparent landmark are used, other limitations of this study include cases where non‐standard indexing is used. This can occur for good reasons including taller patients who may require alternate positions on the table due to limits in table motion. Furthermore, while this study can help determine the expected accuracy of patient setup in terms of table coordinates, the data here are specific to the first patient setup alone and do not reflect the fraction‐to‐fraction variation in table coordinates. Therefore, no recommendations of the tolerance for subsequent treatment fractions can be made with this data.

## 5 CONCLUSIONS

Prediction of couch coordinates is achievable with minimal additional steps required in the simulation process. The vertical dimension was predicted accurately regardless of site, immobilization device, or treatment machine. The longitudinal dimension required consistent use of table indexing, a procedure commonly practiced by radiation therapy clinics. Laterally, the predictions were accurate, but had larger uncertainty in cases where sidedness dictated a laterally off‐center patient position. Action levels at or around 2 cm might be appropriate for the practice described in this study, and the use of this prediction tool can be used to improve patient safety by detecting setup errors before any sort of ionization radiation is used for verification imaging or treatment.

## CONFLICTS OF INTEREST

The authors have no other relevant conflicts of interest to disclose.
